# Exploring the conformational dynamics and thermodynamics of *EGFR* S768I and G719X + S768I mutations in non-small cell lung cancer: An *in silico* approaches

**DOI:** 10.1515/biol-2022-0768

**Published:** 2023-11-27

**Authors:** Jun-Ling Wang, Ming-Sheng Liu, Yu-Dong Fu, Qiang-Bo Kan, Chun-Yan Li, Rong Ma, Zhe-Wei Fang, Hong-Xia Liu, Meng-Xian Li, Jia-Ling Lv, Peng Sang, Chao Zhang, Hong-Wei Li

**Affiliations:** Clinical Laboratory, Kunming Medical University Affiliated Qujing Hospital, Qujing 655000, China; Department of Urological Surgery, Kunming Medical University Affiliated Qujing Hospital, Qujing 655000, China; Department of Thoracic Surgery, Kunming Medical University Affiliated Qujing Hospital, Qujing 655000, China; Department of Oncology, Kunming Medical University Affiliated Qujing Hospital, Qujing 655000, China; School of Life Science, Dali University, Dali 671003, China

**Keywords:** epidermal growth factor receptor G719X and S768I mutations, non-small cell lung cancer, molecular dynamics simulation, conformation, free energy landscape

## Abstract

Non-small cell lung cancer (NSCLC) is often driven by mutations in the epidermal growth factor receptor (*EGFR*) gene. However, rare mutations such as G719X and S768I lack standard anti-EGFR targeted therapies. Understanding the structural differences between wild-type EGFR and these rare mutants is crucial for developing EGFR-targeted drugs. We performed a systematic analysis using molecular dynamics simulations, essential dynamics (ED), molecular mechanics Poisson–Boltzmann surface area, and free energy calculation methods to compare the kinetic properties, molecular motion, and free energy distribution between wild-type EGFR and the rare mutants’ structures G719X-EGFR, S768I-EGFR, and G719X + S768I-EGFR. Our results showed that S768I-EGFR and G719X + S768I-EGFR have higher global and local conformational flexibility and lower thermal and global structural stability than WT-EGFR. ED analysis revealed different molecular motion patterns between S768I-EGFR, G719X + S768I-EGFR, and WT-EGFR. The A-loop and αC-helix, crucial structural elements related to the active state, showed a tendency toward active state development, providing a molecular mechanism explanation for NSCLC caused by *EGFR* S768I and *EGFR* G719C + S768I mutations. The present study may be helpful in the development of new EGFR-targeted drugs based on the structure of rare mutations. Our findings may aid in developing new targeted treatments for patients with *EGFR* S768I and *EGFR* G719X + S768I mutations.

## Graphical abstract

**Figure:**
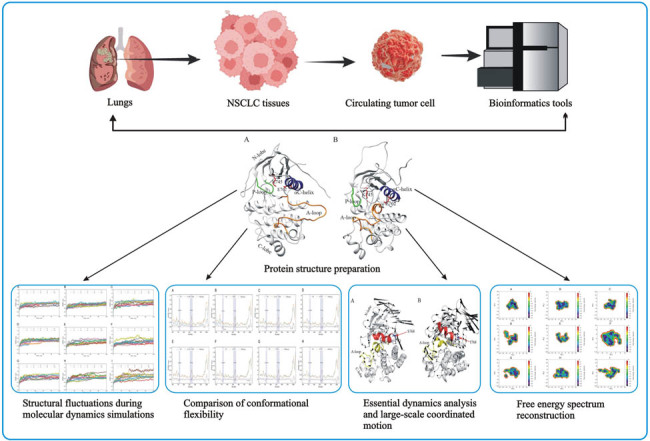

## 1 Background

Lung cancer is the leading cause of death from malignant tumors worldwide [1]. Among lung cancers, non-small cell lung cancer (NSCLC) accounts for 85% of all cases, and epidermal growth factor receptor (*EGFR*) gene mutations have been found in at least 59.4% of Asian NSCLC patients [2]. Among observed *EGFR* mutations in NSCLC patients, approximately 85% are common mutations (*EGFR* 19-Del and L858R), while the remaining 15% are rare mutations [3]. In coal areas, the distribution of *EGFR* mutations is significantly different, with common mutations accounting for 54.41% and rare mutations accounting for 45.59% [4]. According to Amelia et al.’s report [5], mutated *EGFR* results in the constant activation of the tyrosine kinase domain, which in turn promotes the growth, proliferation, invasion, and metastasis of cancer cells without the presence of ligands, making it an essential oncogenic driver and target point for cancer therapy [6].

Numerous EGFR tyrosine kinase domain inhibitors (TKIs) and vascular endothelial growth factor (VEGF)/VEGF receptor (VEGFR) neutralizing antibodies have been developed and approved for the treatment of NSCLC [7]. The FDA has approved EGFR-TKIs in various countries, with three generations available: gefitinib, afatinib, and osimertinib. Compared to chemotherapy, targeted therapy is more specific, sparing normal cells and reducing adverse side effects. It has been observed to significantly alleviate the progression of NSCLC driven by classic *EGFR* mutations (19-Del and L858R) while improving progression-free and overall survival rates [2]. Currently, no effective treatments are available for rare *EGFR* mutations in the treatment of NSCLC. Therefore, developing targeted drugs for rare EGFR mutations has become a crucial focus for researchers to improve patient treatment options.

The structure and function of EGFR protein have proven useful in developing new targeted drugs. Research has shown that EGFR is a transmembrane receptor tyrosine kinase composed of 1,210 amino acids and has a molecular weight of approximately 134 kDa. The protein-coding gene is located in the human chromosome 7p12-14 region. It consists of 28 exons, which include an extracellular ligand-binding domain that has 621 amino acids (AA) (25–645), an α-helical transmembrane domain that has 23 AA (646–668), a cytoplasmic tyrosine kinase domain that has 273 AA (707–979), and a carboxy-terminal (C-terminal) signaling domain that has 229 AA (982–1,210) [8]. The intracellular kinase domain of EGFR is composed of three parts: the amino-terminal (N-terminal) lobe, which can be further divided into a P-loop, an ATP-binding pocket, and an αC-helix; the hinge region (^791^QLMPF^795^) that connects the N-terminal lobe to the C-terminal lobe; and the C-terminal lobe, which includes the Asp-Phe-Gly (DFG) motif, catalytic loop (HRD^837^LAARN), and A-loop [8].

**Figure 1:**
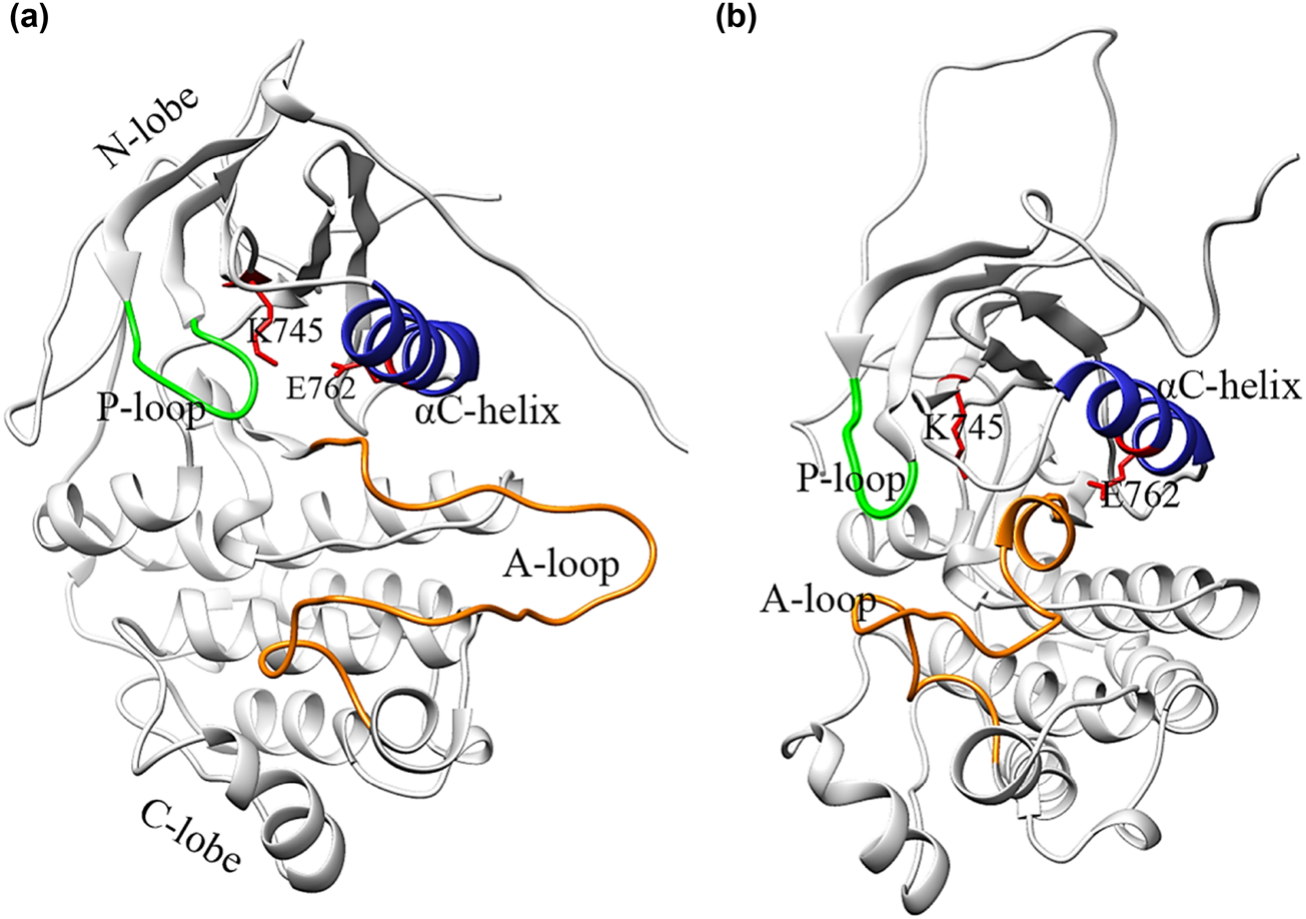
Comparison of active (a) and inactive (b) structures of the kinase domain of EGFR. Key structural elements are colored in blue (αC-helix), green (P-loop), orange (A-loop), and red (residues K745 and E762, shown as sticks), made by Chimera Software.

The ATP-binding cleft is at the interface between the N-lobe and C-lobe [8]. The activation of kinases is primarily regulated through conformational changes of four conserved motifs located near the active site. These motifs include the glycine-rich phosphate-binding loop (P-loop), which controls the αC-helix, the activation loop (A-loop), the DFG motif, and the αC-helix (Figure 1). The active state of EGFR (Figure 1a) is characterized by an extended conformation of the A-loop, which has a hairpin structure in its N-terminal region, an “in” conformation of αC-helix, and features a K745-E762 salt bridge that serves as an anchor for the αC-helix. In the inactive state of EGFR (Figure 1b), a short α-helix is present at the N-terminal region of the A-loop. This short α-helix interacts with the αC-helix, leading to self-inhibitory interactions that stabilize the outward rotation of the αC-helix and maintain an “out” conformation [7]. This conformation prevents the formation of the K745-E762 salt bridge and is similar to the inactive conformation of Rous sarcoma oncogene cellular homolog (Src) and cyclin-dependent kinase 2 [8].

Patients with S768I *EGFR* mutation have poorer prognoses due to shorter median survival and progression-free survival than other *EGFR* mutations [9]. S768 is located in exon 20 of *EGFR*, specifically in the αC-β4 loop. In this location, the hydrophilic serine is replaced by a hydrophobic isoleucine, which has greater steric resistance than the previous serine, resulting in stronger hydrophobic interactions between the αC-helix and the adjacent β9 chain. This enhances the effect and stabilizes the enthalpy of the active conformation of “αC-in.” G719 is in the N-terminal lobe and represents the initial glycine in the P-loop’s widely preserved “GXGXXG” motif. When non-glycine residues (A, C, D, S) replace G719, it leads to a substantial decrease in the flexibility of the P-loop, which weakens the inhibition of the hydrophobic interaction of αC-helix in the inactive configuration and destabilizes the inactive conformation, thus leading to the active conformation.

According to the structure analysis by Du et al. [10], both G719X and S768I mutations were categorized as P-loop and αC-helix compression mutations, and G719X + S768I is the most prevalent subtype among rare compound mutations of *EGFR* [11]. Wang et al.’s research in 2022 found that a significant number of NSCLC lung cancer patients in the high-incidence area of rural lung cancer in the coal-producing region of eastern Yunnan in southwest China carry the compound mutation of G719X + S768I, making up 16.67% of all *EGFR* mutations.

Furthermore, G719C + S768I accounted for 68.82% of all G719X + S768I mutations [4]. The S768I and G719X + S768I mutations may change the adjacent structure around the mutated amino acid, such as a reduction in hydrogen bonding, leading to changes in the binding affinity of drug inhibitors to EGFR and poor drug efficacy [12]. The activation mechanism caused by the S768I, G719X, and G719X + S768I mutations remains unclear, and understanding the molecular basis of the differences between these rare mutants and the wild type is crucial for developing new targeted treatment strategies.

Therefore, this study aims to provide an overview of EGFR mutations in NSCLC, emphasizing their prevalence and the distinction between common and rare mutations while highlighting the significance of targeted therapies over chemotherapy. Our primary research focus was to comprehensively analyze the kinetic and thermodynamic behavior of EGFR mutations, specifically S768I, G719X, and G719X + S768I, in comparison to wild-type EGFR, using molecular dynamics (MD) simulations and free energy mapping. This analysis seeks to elucidate these mutants’ conformational flexibility, kinetic properties, molecular motion, and free energy distribution, shedding light on their role in NSCLC and contributing to developing more effective targeted treatments.

Various computational models were utilized to understand the kinetic mechanism of EGFR mutations, such as geometric properties of EGFR, binding free energy, hydrogen bond analysis, and stability analysis [13]. These models will be used to decode the mechanism of the *EGFR* S768I and G719X + S768I mutations. Through MD simulations and the reconstruction of free energy maps, we sought to answer the following key questions:

1. How do the conformational flexibility and kinetic properties of S768I-EGFR, G719X, and G719X + S768I-EGFR differ from those of WT-EGFR, and what implications do these differences have for the activation state of the protein?
2. What are the distinctive patterns of molecular motion exhibited by these mutant EGFR proteins, and how do they contribute to our understanding of their behavior in the context of NSCLC?
3. What insights into the free energy distribution of these EGFR mutants can be gained, and how can these insights inform the development of targeted treatment strategies for NSCLC patients carrying these rare mutations?

In this study, we conducted a systematic computational study to compare the kinetic and thermodynamic behavior of WT-EGFR, G719X-EGFR, S768I-EGFR, and G719X + S768I-EGFR. Our results showed that S768I-EGFR, G719X, and G719X + S768I-EGFR exhibited higher conformational flexibility and a greater tendency to transition to the active state than WT-EGFR. These findings provide a molecular mechanism explanation for the *EGFR* S768I, G719X, and G719X + S768I mutation leading to NSCLC and can help in developing new targeted treatment strategies for patients with these rare mutations.

## 2 Materials and methods

### 2.1 Protein structure preparation

The structure of the inactive wild-type EGFR was downloaded from the Protein Data Bank (PDB; https://www.rcsb.org) database (PDB code: 2GS7). The missing residues were modeled using MODELER [14], and the mutants (S768I-EGFR, G719X, and G719X + S768I-EGFR) were prepared using PYMOL [15].

### 2.2 MD simulation

MD simulation is a computational method used to study the equilibrium state distribution, state-to-state transitions, and dynamic behavior of biomolecules [16]. In this study, we employed the GROMACS-20.6 software package for MD simulations. The starting structures for the simulations were the WT-EGFR, G719X-EGFR, S768I-EGFR, and G719X + S768I-EGFR models. The AMBER99SB-LIDN force field [17,18] was utilized, along with the TIP3P water model [19]. Periodic boundary conditions were implemented in a dodecahedral box, with a minimum distance of 1.0 nm between the protein and the box wall. Water molecules were added to fill the simulation box to ensure a physiological salt concentration of 150 mM, and Na and Cl ions were introduced. The systems underwent global energy minimization using the steepest descent algorithm to resolve contacts and conflicts. Before the MD production simulation, a 100 ns equilibration phase was conducted in the NVT and NPT ensembles to allow for sufficient interaction between the solute and solvent. The MD simulations were performed with a time step of 2 fs, and bond lengths were constrained using the LINCS algorithm [20]. Long-range electrostatic interactions were handled using the Particle Mesh Ewald method [21] with a cutoff of 1.0 nm.

In comparison, van der Waals interactions were computed using a twin-range cutoff scheme of 1.0 nm (short-range) and 1.4 nm (long-range). The solute and solvent temperature were maintained at 300 K using a coupling time constant τt of 0.1 ps [22]. The pressure was controlled at 1 atm using a Parrinello–Rahman barostat [23,24] with a coupling time constant τp of 0.5 ps. To enhance conformational sampling, a multi-replica strategy was employed. Each system underwent ten independent 100 ns production MD simulations, with atoms assigned different initial velocities sampled from a Maxwell–Boltzmann distribution at 300 K. The simulations were carried out at a constant temperature of 300 K and pressure of 1 atm for 100 ns.

### 2.3 Dynamic performance analysis

Root mean square deviation (RMSD) and Cα root mean square fluctuation (RMSF) were calculated using the GROMACS tools “gmx rmsd” and “gmx rmsf,” respectively [17].

### 2.4 Essential dynamics (ED) analysis

For ED analysis in this study, we utilized principal component analysis [25], a widely used mathematical dimensionality reduction method that extracts the most critical change patterns from variables. To construct and diagonalize the Cα atomic covariance matrix, we utilized the “gmx covar” tool. Trajectories were then projected onto the eigenvectors using the “gmx anaeig” tool. The modevectors.py script in PYMOL was used to obtain porcupine plots that display the extreme structure of a certain eigenvector.

### 2.5 Free energy map reconstruction

The free energy landscape (FEL) was used to characterize the thermodynamic and energetic properties of WT-EGFR, G719X-EGFR, S768I-EGFR, and G719X + S768I-EGFR. The GROMACS-20.6 software package was used to perform MD simulations for both systems, and the ED analysis was carried out to obtain the first few eigenvectors. The two-dimensional preparatory subspace formed along eigenvectors 1 and 2 was used as the reaction coordinates to reconstruct the FEL of the two systems. The global free energy minimization region was characterized by different sizes and depths of free energy wells, indicating a rugged and rough free energy surface.

## 3 Results

### 3.1 Structural fluctuations during MD simulations

**Figure 2:**
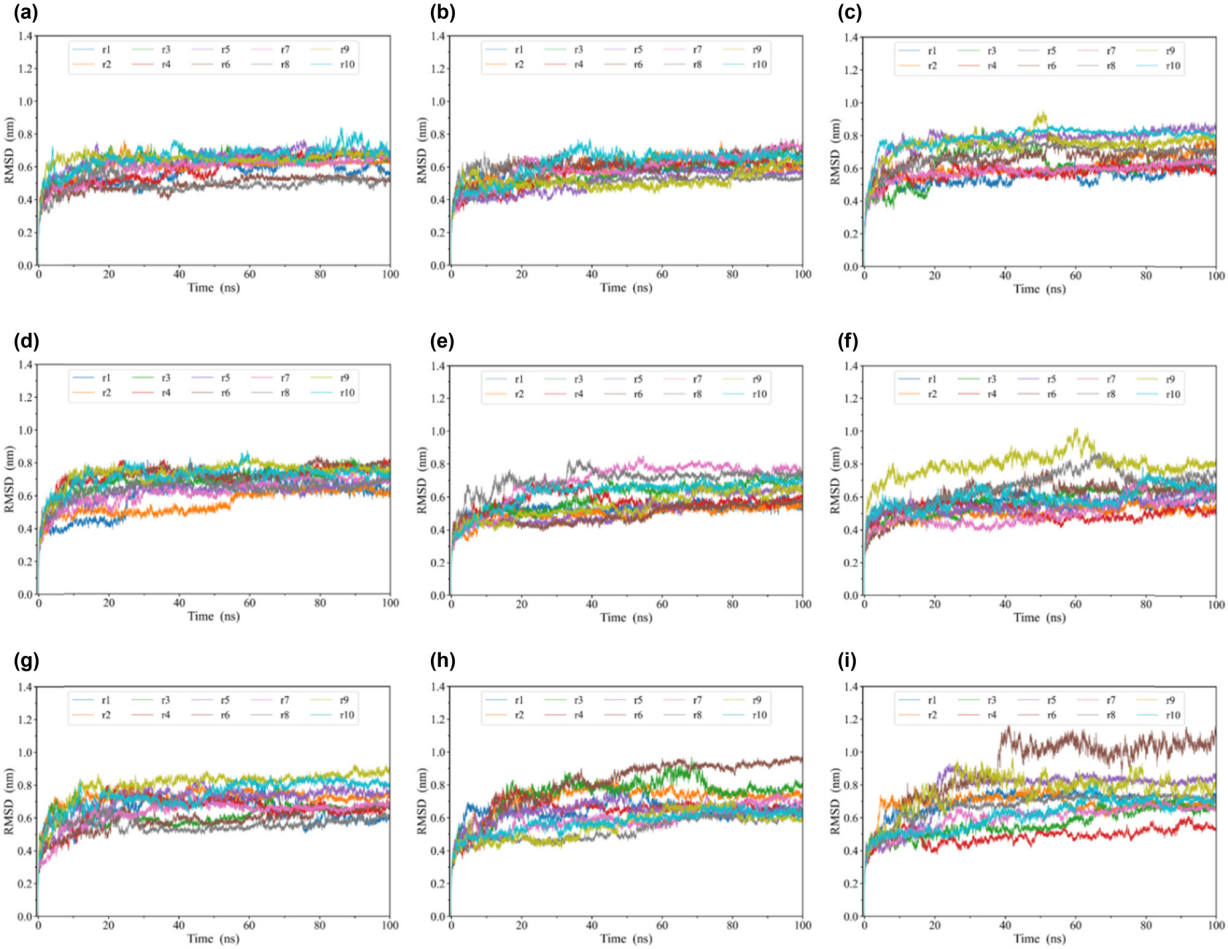
RMSD values of the backbone atoms of G719C-EGFR (a), G719A-EGFR (b), WT-EGFR (c), G719A + S768I-EGFR (d), G719S-EGFR (e), G719C + S768I-EGFR (f), S768I-EGFR (g), G719D-EGFR (h), and G719D + S768IS-EGFR (i) relative to the initial structure as a function of time. r1–r10 represents ten independent copies of MD simulations.

To evaluate the stability of the structures of WT-EGFR, G719X-EGFR, S768I-EGFR, and G719X + S768I-EGFR during simulation, we calculated the RMSD values of the backbone atoms for each simulation replica concerning the initial structure (Figure 2). The RMSD curves of ten analog copies of G719C-EGFR and G719A-EGFR reached relatively stable RMSD fluctuations in 5 and 6 ns, respectively (Figure 2a and b). The RMSD curves of ten simulated copies of WT-EGFR and G719A + S768I-EGFR each required 10 ns to reach relatively stable fluctuations (Figure 2c and d). However, the ten analog copies of G719S-EGFR, G719C + S768I-EGFR, S768I-EGFR, G719D-EGFR, and G719D + S768I-EGFR required 12, 12, 15, 22, and 23 ns, respectively, to reach relatively stable RMSD fluctuations (Figure 2e–i).

These results indicate that G719C-EGFR and G719A-EGFR are easier to achieve equilibrium fluctuations than WT-EGFR. The time required for G719A + S768I-EGFR and WT-EGFR to reach equilibrium fluctuations is similar. G719S-EGFR, G719C + S768I-EGFR, S768I-EGFR, G719D-EGFR, and G719D + S768I-EGFR are harder to achieve equilibrium fluctuations than WT-EGFR. In other words, the structural stability of G719A + S768I-EGFR is similar to that of WT-EGFR, and the structural stability of G719C-EGFR and G719A-EGFR is higher than that of WT-EGFR. Additionally, the structural stability of G719S-EGFR, G719C + S768I-EGFR, S768I-EGFR, G719D-EGFR, and G719D + S768I-EGFR is lower than that of WT-EGFR. Therefore, the lower the structural stability, the higher the ability of the protein to undergo conformational changes.

These findings indicate increased structural dynamics in these mutants, which may have important implications for their functional properties. In particular, the elevated conformational flexibility of the A-loop region, associated with the active state of EGFR, suggests that these mutants may have an enhanced capacity to transition from an inactive to an active conformation. This insight is crucial for understanding the potential oncogenic properties of these mutants in NSCLC, where EGFR dysregulation is a vital driver of tumorigenesis. This observation suggests that these mutants may have a heightened propensity to adopt functionally relevant states, including the active conformation. Clinical implications arise as these mutants could drive aggressive NSCLC phenotypes and respond differently to targeted therapies.

### 3.2 Comparison of conformational flexibility

**Figure 3:**
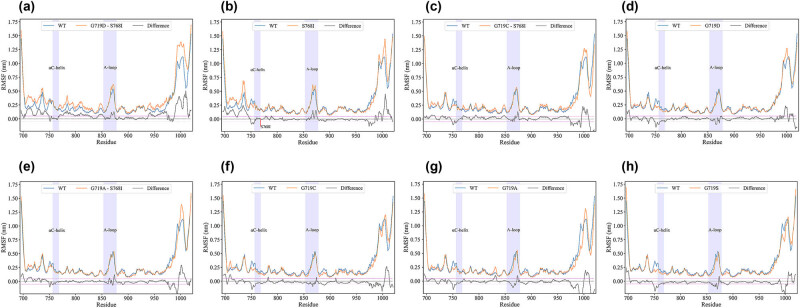
Cα atom RMSF value WT-EGFR (blue line) and G719D + S768I-EGFR (a), S768I-EGFR (b), G719C + S768I-EGFR (c), G719D-EGFR (d), G719A + S768I-EGFR (e), G719C-EGFR (f), G719A-EGFR (g), G719S-EGFR (h) (orange line) as a function of residue number. The RMSF difference between S768I-EGFR and WT-EGFR is the black line. Highlight the αC-helix and A-loop with a light purple shade.

To ensure that the calculated RMSF values accurately reflect the natural conformational flexibility, we observed Figure 2. We found that the fluctuations between EGFR-WT and EGFR-MT (Mutant Type) become relatively stable after 20 ns. As a result, we selected the 20–100 ns trajectories of EGFR-WT and EGFR-MT for subsequent analysis and combined each simulated copy’s balanced trace (20–100 ns) in a series to form an 800 ns long balanced trace. We calculated the RMSF value of the Cα atom based on the connecting series equilibrium tracks of G719D + S768I-EGFR, S768I-EGFR, G719C + S768I-EGFR, G719D-EGFR, G719A + S768I-EGFR, WT-EGFR, G719C-EGFR, G719A-EGFR, and G719S-EGFR. The RMSF value is often used to index the protein’s structural flexibility (Figure 3). The average RMSF values of the Cα atom for G719D + S768I-EGFR, S768I-EGFR, G719C + S768I-EGFR, G719D-EGFR, G719A + S768I-EGFR, WT-EGFR, G719C-EGFR, G719A-EGFR, and G719S-EGFR were 0.362, 0.312, 0.294, 0.289, 0.288, 0.287, 0.270, 0.268, and 0.267 nm, respectively (Table 1). This indicates that compared to WT-EGFR, G719D + S768I-EGFR, S768I-EGFR, G719C + S768I-EGFR, G719D-EGFR, and G719A + S768I-EGFR have higher global conformational flexibility.

**Table 1:** Average RMSF values of Cα atoms

	G719D + S768I	S768I	G719C + S768I	G719D	G719A + S768I	WT	G719C	G719A	G719S
RMSF (average)	0.361892	0.311604	0.293938	0.289211	0.288043	0.286596	0.269909	0.26771	0.266783

Upon careful examination of the RMSF diagram (Figure 3), it was observed that G719D + S768I-EGFR, S768I-EGFR, G719C + S768I-EGFR, G719D-EGFR, and G719A + S768I-EGFR exhibit higher RMSF values than WT-EGFR in certain structural regions. To quantify these differences, the RMSF values of G719D + S768I-EGFR, S768I-EGFR, G719C + S768I-EGFR, G719D-EGFR, and G719A + S768I-EGFR were subtracted from the RMSF value of WT-EGFR at the corresponding residue positions (black curve in Figure 3). The results revealed that these mutant proteins have higher conformational flexibility (RMSF difference > 0) in multiple regions, including the A-loop region that is associated with the active state of EGFR, which involves the rearrangement of the A-loop, and the higher conformational flexibility of the A-loop in these mutant proteins may facilitate the transition of inactivated EGFR to the activated state.

Moreover, our ED analysis revealed that certain mutants experience larger structural fluctuations and conformational changes than WT-EGFR, implying the existence of distinct dynamic behavior that may be exploited in drug design. These mutants could be more susceptible to compounds that selectively stabilize or disrupt specific conformations, opening up new avenues for drug development strategies tailored to individual EGFR mutations. The increased conformational diversity observed in certain mutants opens new avenues for precision medicine. Targeted therapies that specifically exploit the unique structural dynamics of EGFR mutants may provide a more effective treatment strategy for patients. For example, drugs designed to stabilize or disrupt specific conformations associated with mutant EGFR variants could improve treatment outcomes. Our study identifies potential vulnerabilities in these mutants that can be targeted with tailored therapeutic interventions.

### 3.3 ED analysis and large-scale coordinated motion

The ED analysis based on the MD simulation trajectory was used to extract the protein’s most important or largest scale motion mode in the multi-dimensional conformational space. The eigenvectors and eigenvalues of wild-type and mutant EGFR were obtained by analyzing the ED of their respective tandem equilibrium trajectories.

The total mean square fluctuation of Cα, also known as TMSF (the sum of eigenvalues of all eigenvectors), was calculated for G719D + S768I-EGFR, S768I-EGFR, G719A + S768I-EGFR, G719D-EGFR, G719C + S768I-EGFR, WT-EGFR, G719S-EGFR, G719C-EGFR, and G719A-EGFR. The TMSF values were 75.507, 61.299, 52.804, 51.522, 49.712, 49.196, 47.750, 47.507, and 43.594 nm^2^, respectively (Table 2). These values indicated that G719D + S768I-EGFR, S768I-EGFR, G719A + S768I-EGFR, G719D-EGFR, and G719C + S768I-EGFR experienced more drastic structural fluctuations or conformational changes than WT-EGFR, G719S-EGFR, G719C-EGFR, and G719A-EGFR during the simulation, which is consistent with the comparative analysis results based on RMSD and RMSF.

**Table 2:** Total mean square fluctuation of Cα in WT-EGFR and S768I-EGFR

	G719D + S768I	S768I	G719A + S768I	G719D	G719C + S768I	WT	G719S	G719C	G719A
TMSF	75.5066	61.29884	52.80355	51.52172	49.71187	49.19613	47.75026	47.50714	43.59395

**Figure 4:**
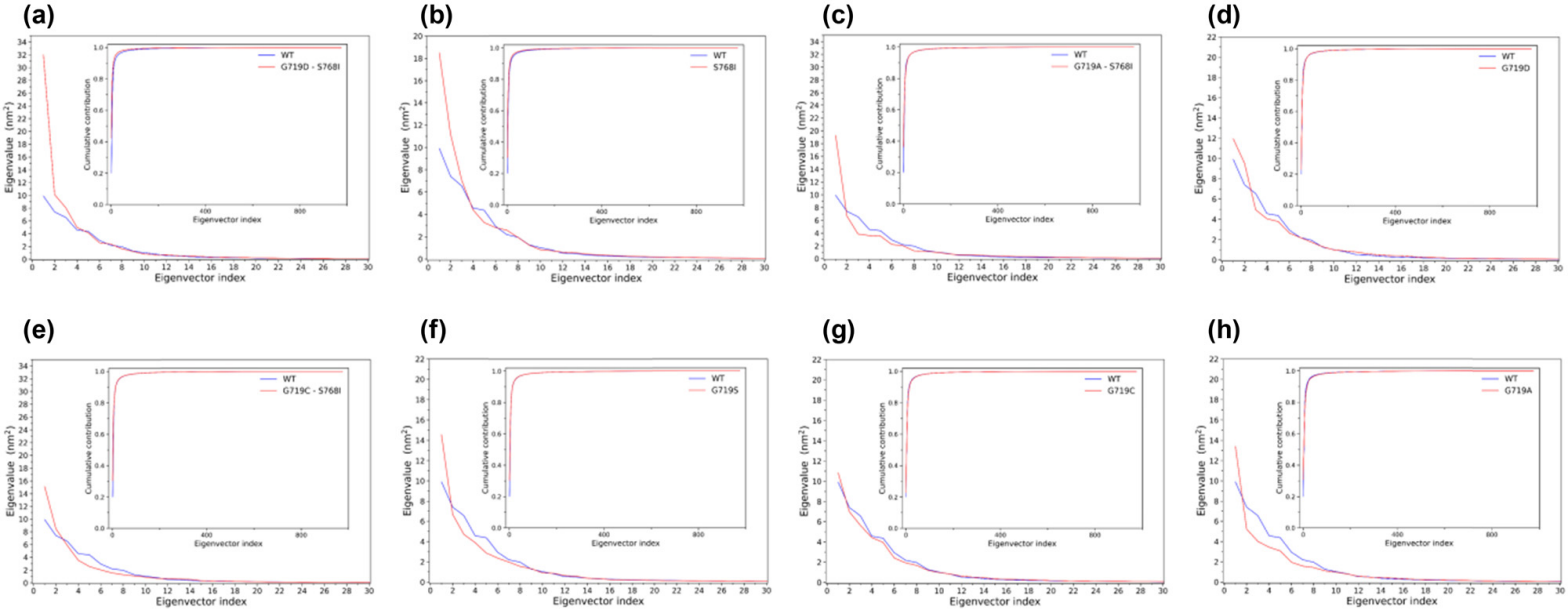
The first 30 eigenvectors eigenvalues of G719D + S768I-EGFR (a), S768I-EGFR (b), G719A + S768I-EGFR (c), G719D-EGFR (d), G719C + S768I-EGFR (e), G719S-EGFR (f), G719C-EGFR (g), and G719A-EGFR (h) (main plot) and cumulative contributions to the TMSF for all eigenvectors (inset), made by python script.

The top 30 eigenvalues of the eigenvectors of G719D + S768I-EGFR, S768I-EGFR, G719A + S768I-EGFR, G719D-EGFR, G719C + S768I-EGFR, WT-EGFR, G719S-EGFR, G719C-EGFR, and G719A-EGFR are shown in Figure 4. The inset in the figure shows the cumulative contribution of all eigenvectors to the TMSF value. The first eigenvectors of G719D + S768I-EGFR, S768I-EGFR, G719A + S768I-EGFR, and G719D-EGFR all had larger eigenvalues than WT-EGFR. As the number of eigenvectors increased, the eigenvalues decreased rapidly. Until the tenth eigenvector afterward, the change in the eigenvalues tended to be gentle. The eigenvalues of the first eigenvectors of G719D + S768I-EGFR, S768I-EGFR, G719A + S768I-EGFR, G719D-EGFR, G719C + S768I-EGFR, G719S-EGFR, G719C-EGFR, and G719A-EGFR were significantly higher than those of WT-EGFR, indicating that G719D + S768I-EGFR, S768I-EGFR, G719A + S768I-EGFR, G719D-EGFR, G719C + S768I-EGFR, G719S-EGFR, G719C-EGFR, and G719A-EGFR moved more violently than WT-EGFR along the first eigenvector.

The ED analysis revealed that the eigenvalues of the first eigenvectors of G719D + S768I-EGFR, S768I-EGFR, G719A + S768I-EGFR, G719D-EGFR, G719C + S768I-EGFR, G719S-EGFR, G719C-EGFR, and G719A-EGFR were significantly higher than those of WT-EGFR, indicating that these mutants experienced more drastic structural fluctuations or conformational changes than WT-EGFR along the first eigenvector. The cumulative contributions of the first two eigenvectors of WT-EGFR, S768I-EGFR, and G719C + S768I-EGFR to TMSF were 35.2, 38.1, and 37.3%, respectively, while the cumulative contributions of the first ten eigenvectors were 78.9, 79.5, and 79.6%, respectively. These results suggest that the essential subspace formed by the first two eigenvectors contains the most significant collective motion and the most dominant conformational states or substrates obtained by MD simulation sampling.

Furthermore, the first eigenvectors of G719D + S768I-EGFR, S768I-EGFR, G719A + S768I-EGFR, G719D-EGFR, and G719C + S768I-EGFR had larger eigenvalues than WT-EGFR, indicating that these mutants had larger motion amplitude or conformational freedom degree than WT-EGFR along the first eigenvector. In such a high-dimensional conformational space, only the first ten eigenvectors contributed more than 80% to the TMSF (overall conformational fluctuation), indicating that our intrinsic kinetic analysis successfully extracted the most dominant movement patterns of EGFR. Overall, compared with WT-EGFR, G719D + S768I-EGFR, S768I-EGFR, G719A + S768I-EGFR, G719D-EGFR, and G719C + S768I-EGFR required a smaller number of eigenvectors to reach the same level of cumulative contribution.

**Figure 5:**
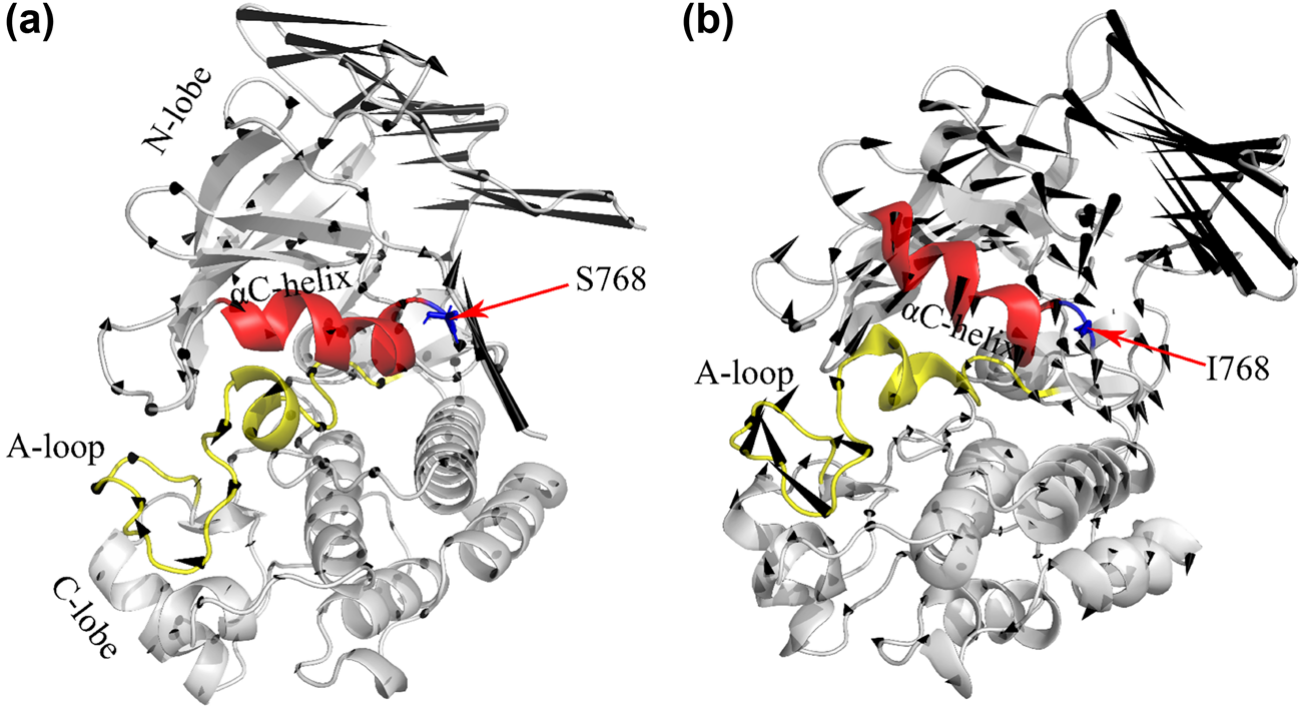
Glitch plots of WT-EGFR (a) and S768I-EGFR (b) made according to the first eigenvector projection. Where the cone is drawn on the Cα atom, its orientation and length ratio represents the motion direction and Cα fluctuation amplitude made by PYMOL.

The motion pattern of the two EGFRs along the first eigenvector is shown in a glitch diagram (Figure 5). The first eigenvector, having the largest eigenvalue, is considered the largest-amplitude collective motion or the most significant motion pattern. Figure 5 shows that the S768I mutation causes the N-lobe of EGFR to exhibit a more considerable displacement than WT-EGFR. Careful observation revealed that the A-loop and αC-helix of S768I-EGFR had larger collective movement displacements than WT-EGFR. Additionally, the C-terminal loop region of the A-loop of S768I-EGFR showed a hairpin conformational change in movement amplitude. The N-terminal short α-helix conformation of the A-loop also changed significantly, appearing to unwind.

Moreover, the movement direction of the αC-helix, another crucial structural element related to EGFR activation, was downward and inward, opposite to the outward rotation of αC-helix “out” conformation. The αC-helix tended to develop toward an “in” conformation trend. These conformational change trends of A-loop and αC-helix provide an essential basis for the transition of EGFR from an inactive to an active conformation.

### 3.4 Free energy spectrum reconstruction

**Figure 6:**
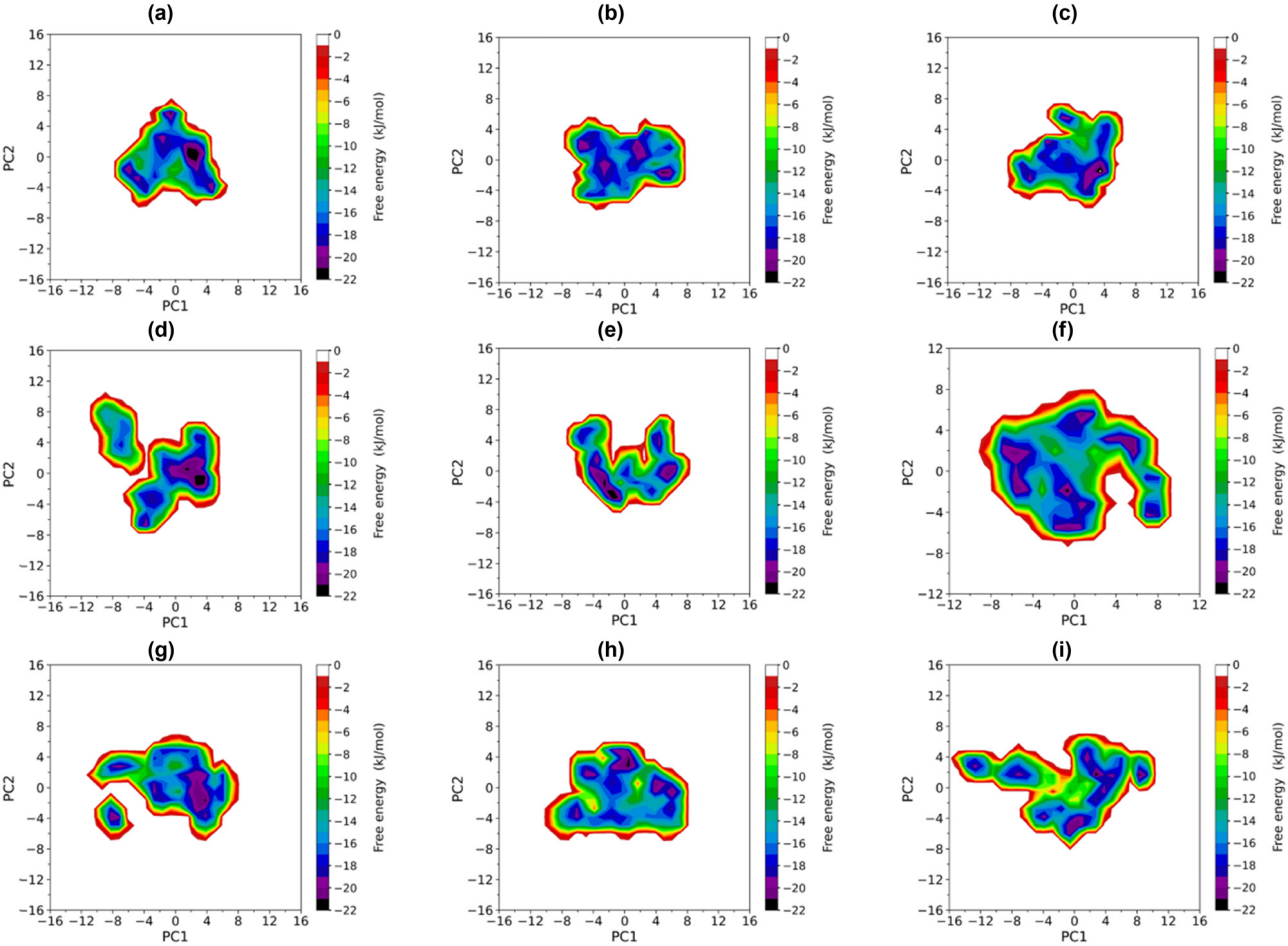
Free energy spectrum (FEL) of WT-EGFR (a), G719A-EGFR (b), G719C-EGFR (c), G719D-EGFR (d), G719S-EGFR (e), S768I-EGFR (f), G719A + S768I-EGFR (g), G7A9C + S768I-EGFR (h), and G719D-EGFR (i) with the projection of eigenvector 1 and eigenvector 2 as the reaction coordinates, the color bar on the right represents the free energy of FEL energy value (accomplished through the use of a Python script).

The FEL of WT-EGFR, G719A-EGFR, G719C-EGFR, G719D-EGFR, G719S-EGFR, S768I-EGFR, G719A + S768I-EGFR, G719C + S768I-EGFR, and G719D-EGFR were reconstructed using the first and second eigenvector projections as reaction coordinates (Figure 6). Analysis of the FEL of WT-EGFR and S768I-EGFR shows that the FEL of WT-EGFR on PC1 and PC2 spans −6.9 to 7.8 nm and −8.0 to 6.8 nm, respectively, forming a triangular, regular, and continuous shape. However, the FEL of S768I-EGFR on PC1 and PC2 spans −7.5 to 7.9 nm −9.2 to 9.3 nm, respectively, displaying an irregular and divergent shape. The FEL of S768I-EGFR occupies a larger area than WT-EGFR’s in the conformational subspace formed by the first two eigenvectors. Additionally, the FEL of WT-EGFR contains six free energy wells (free energy value ≤−19 kJ/mol), while that of S768I-EGFR contains eight free energy wells, indicating that S768I-EGFR has more conformational diversity than WT-EGFR.

Compared with S768I-EGFR, WT-EGFR has two larger free energy wells (≤−21 kJ/mol), implying higher thermal stability. Greater conformational diversity signifies higher conformational freedom or structural variability, allowing S768I-EGFR to sample more conformational states or substrates. However, the increased degree of conformational freedom also leads to decreased thermal and structural stability of S768I-EGFR. Therefore, S768I-EGFR has greater conformational diversity and more complex dynamic behavior than WT-EGFR, with an improved ability to approach the active state.

## 4 Discussion

Activating wild-type EGFR requires ligand binding, which induces a conformational change promoting receptor homodimerization or heterodimerization with other EGFR family members. This event leads to the autophosphorylation of tyrosine residues in the EGFR tail and the activation of several signaling pathways [26]. These pathways coordinate essential cellular processes, such as cell differentiation, proliferation, and migration. Previous studies have shown that the L858R mutation in *EGFR* disrupts its inactive form, leading to an equilibrium shift toward the active state. This shift enables the receptor to activate independently of ligand binding [27]. The conformational change from inactive to active state is directly linked to EGFR’s transition. However, the molecular mechanism of how the *EGFR* S768I and G719X + S768I mutations affect the structure of EGFR and its relation to NSCLC remains unclear. Understanding the molecular basis of the conformational distribution of the S768I and G719X + S768I mutants of EGFR is vital for in-depth research into the molecular mechanisms of mutation-activated NSCLC and related drug development. In this study, we conducted long-term MD simulations of S768I-EGFR, G719X + S768I-EGFR, and WT-EGFR to investigate the structural and kinetic differences between the rare mutants and wild type.

The RMSF analysis results of S768I-EGFR, G719X + S768I-EGFR, and WT-EGFR indicated that S768I-EGFR and G719D + S768I-EGFR had greater global and local conformational flexibility than WT-EGFR, suggesting that the latter had more enthalpy or entropy factors that positively contributed to its structural stability, consistent with previous EGFR structural analyses of L858R and T790M mutations [28]. The study of the local conformational flexibility of the two revealed significantly higher conformational flexibility in the A-loop of S768I-EGFR and G719D + S768I-EGFR. Since the movement ability of the A-loop is critical for the transition of EGFR from an inactive to an active state, the high conformational flexibility of the A-loop would facilitate the transition of EGFR to an active state. The free energy map of S768I-EGFR, G719A + S768I-EGFR, G719C + S768I-EGFR, and G719D-EGFR also demonstrated a more extensive free energy surface and more free energy wells, implying a higher degree of conformational freedom or more complex structural variability, indicating that S768I-EGFR, G719A + S768I-EGFR, G719C + S768I-EGFR, and G719D-EGFR had lower thermal and global structural stability. The greater conformational flexibility, lower thermal stability, and more complex structural diversity of S768I-EGFR, G719A + S768I-EGFR, G719C + S768I-EGFR, and G719D-EGFR improved the ability of EGFR to move toward the active state.

Thus, without ligand induction, S768I-EGFR, G719A + S768I-EGFR, G719C + S768I-EGFR, and G719D-EGFR could reach the active state through a conformational selection mechanism. In contrast, due to its weak conformational flexibility and high conformational rigidity, WT-EGFR must be combined with the ligand to promote further conformational change. Otherwise, it could not reach the active state.

The ED analysis of S768I-EGFR, G719X + S768I-EGFR, and WT-EGFR revealed that S768I-EGFR and G719X + S768I-EGFR have a greater degree of conformational freedom, confirming the previous findings. A comparison of their large-scale collective molecular motions revealed that S768I-EGFR and G719X + S768I-EGFR have more substructures and larger collective displacement amplitudes. These motion direction and amplitude differences lead to distinct conformational changes, particularly in the A-loop and αC-helix of S768I-EGFR and G719D + S768I-EGFR, which tend to transition to the active conformation. The transition of the A-loop and αC-helix between active and inactive states is critical for EGFR regulation, and mutations associated with cancer dysregulate these conformations. The short α-helix of the A-loop interacts with αC-helix autoinhibition to stabilize the “out” conformation of αC-helix, so the conformational change of the short αC-helix will alter its autoinhibitory interaction with αC-helix, promoting the development of αC-helix toward the “in” conformation. Notably, the S768I and G719X + S768I mutations induce more significant molecular movement in the N-terminus, suggesting that the mutation changes the adjacent conformation around the mutant amino acid, leading to differences in overall and local flexibility between S768I-EGFR, G719X + S768I-EGFR, and WT-EGFR [5].

## 5 Limitations

Limited research has been conducted on G719X + S768I by MD. However, Liu et al. utilized the MD method to investigate. They discovered that the G719C mutation impacted the binding free energy of EGFR mutants to gefitinib compared to the classical mutation [29]. Chakraborty et al. investigated the G719S-T790M double mutation crucial to gefitinib using 50 ns MD simulation and molecular docking techniques. The researchers noted a greater separation between the P-loop and functional loop in the T790M mutation compared to G719S.

Additionally, they found that the G719S mutation brings the ligand closer to the hinge region, while the T790M mutation moves the ligand out of the binding pocket [30]. This may be one of the reasons for the resistance of the T790M mutant to the first and second-generation EGFR-TKIs. According to the findings of this study, the G719C + S768I RSMF exhibited a relatively larger size and greater conformational flexibility when compared to the G719C, as indicated in Table 2. Clinical studies have demonstrated that gefitinib, a first-generation EGFR-TKI, is more effective in treating G719X than G719X + S768I. Conversely, afatinib, a second-generation EGFR-TKI, has better efficacy against G719X + S768I than G719X, but both have poorer effects on S768I [31]. MD simulations suggest that changes in the binding force between the G719C + S768I-EGFR mutant and EGFR-TKIs may explain the varying efficacy observed. Thus, our findings contribute to the knowledge base by shedding light on the dynamic structural changes that may underlie resistance mechanisms. This knowledge is essential for designing next-generation inhibitors and combination therapies to overcome resistance and extend the benefits of targeted treatment for patients.

## 6 Conclusion and future directions

In conclusion, our study illuminates the dynamic structural characteristics of various EGFR mutants, shedding light on their conformational flexibility and distinct behaviors. Notably, the G719X + S768I mutant, which has been relatively underexplored in MD studies, exhibits remarkable conformational flexibility and structural changes, potentially contributing to its resistance to EGFR-targeted therapies. These findings underscore the importance of precision medicine approaches tailored to specific EGFR mutations. Future research directions should encompass in-depth investigations into the dynamic behaviors of less-studied mutants, developing next-generation inhibitors, and clinical studies to optimize personalized treatment strategies.

Building upon the insights derived from this study, several promising avenues for future research and clinical applications emerge. First and foremost, further investigations are warranted to explore the dynamic behaviors and structural characteristics of EGFR mutants, especially the less-studied G719X + S768I variant, using advanced computational methods and experimental validation. Understanding the distinct conformational landscapes of these mutants can facilitate the development of novel targeted therapies that specifically exploit their structural vulnerabilities. Moreover, the findings hint at the potential for rational drug design targeting the altered binding forces between the mutant EGFR and existing EGFR-TKIs. Future research can delve into developing next-generation inhibitors and combination therapies designed to tackle EGFR resistance mechanisms effectively, ultimately improving patient treatment outcomes. Additionally, clinical studies focusing on the efficacy of tailored treatments based on specific EGFR mutation profiles, as highlighted in our research, can provide valuable insights into personalized medicine approaches for NSCLC patients. Overall, the future directions of this study lie in leveraging these structural insights to advance the understanding of EGFR-related cancers and to develop more precise and effective therapeutic strategies.
